# TRPV2 Regulates Function of Human Neutrophil Granulocytes

**DOI:** 10.1096/fj.202501585RR

**Published:** 2025-09-15

**Authors:** Anna S. Heinemann, Tabea C. Fricke, Christine Herzog, Frank G. Echtermeyer, Andreas Leffler

**Affiliations:** ^1^ Department of Anesthesiology and Intensive Care Medicine Hannover Medical School Hannover Germany

**Keywords:** cytokine, innate immunity, migration, neutrophils, TRPV2

## Abstract

Neutrophils play a vital role in human immune defense, and their dysregulation can cause collateral damage in sepsis and autoimmune diseases. We provide evidence for the involvement of the transient receptor potential vanilloid 2 (TRPV2) in cytokine expression and transmigration in human neutrophils as well as in HL60 cells. These data identify TRPV2 as a candidate target for novel therapeutics regulating neutrophil activity.

## Graphical abstract

In this in vitro study, isolated human neutrophils and differentiated neutrophil‐like HL60 cells were studied to confirm stable expression of the transient receptor potential vanilloid 2 (TRPV2). Patch clamp experiments confirmed channel activity in neutrophils. Furthermore, the relevance of TRPV2 on cell functions like transmigration and cytokine expression was determined, as inhibition of TRPV2 with a selective antagonist decreased transmigration and expression of TNF‐α and IL‐8. These findings suggest TRPV2 as a novel candidate to modify neutrophil activity.

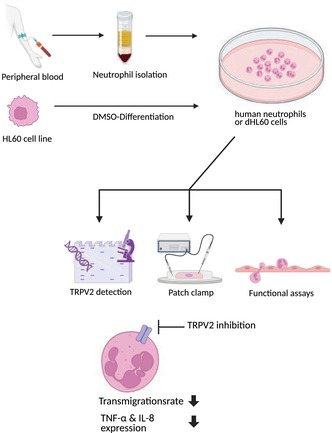

## 1 Introduction

Neutrophils are irreplaceable for effective innate immunity. Defects of neutrophils impair infection resolution, and strong neutrophil activation can aggravate the course of disease. Identifying “druggable” mechanisms that regulate neutrophil activity might aid the development of pharmacological strategies for tuning the immune system. Neutrophils are regulated by several ion channels, including TRPM2, which is sensitive to heat and reactive oxygen species (ROS) [1]. TRPV2 is also heat‐ and ROS‐sensitive, and it was demonstrated to regulate the function of immune cells like macrophages [2], mast cells [3] and B‐lymphocytes [4]. Neutrophils seem to express high levels of TRPV2 [5], but its functional significance remains unexplored. Here we provide novel insights into a significant role of TRPV2 for migration and cytokine expression in neutrophils.

## 2 Results and Discussion

**FIGURE 1:**
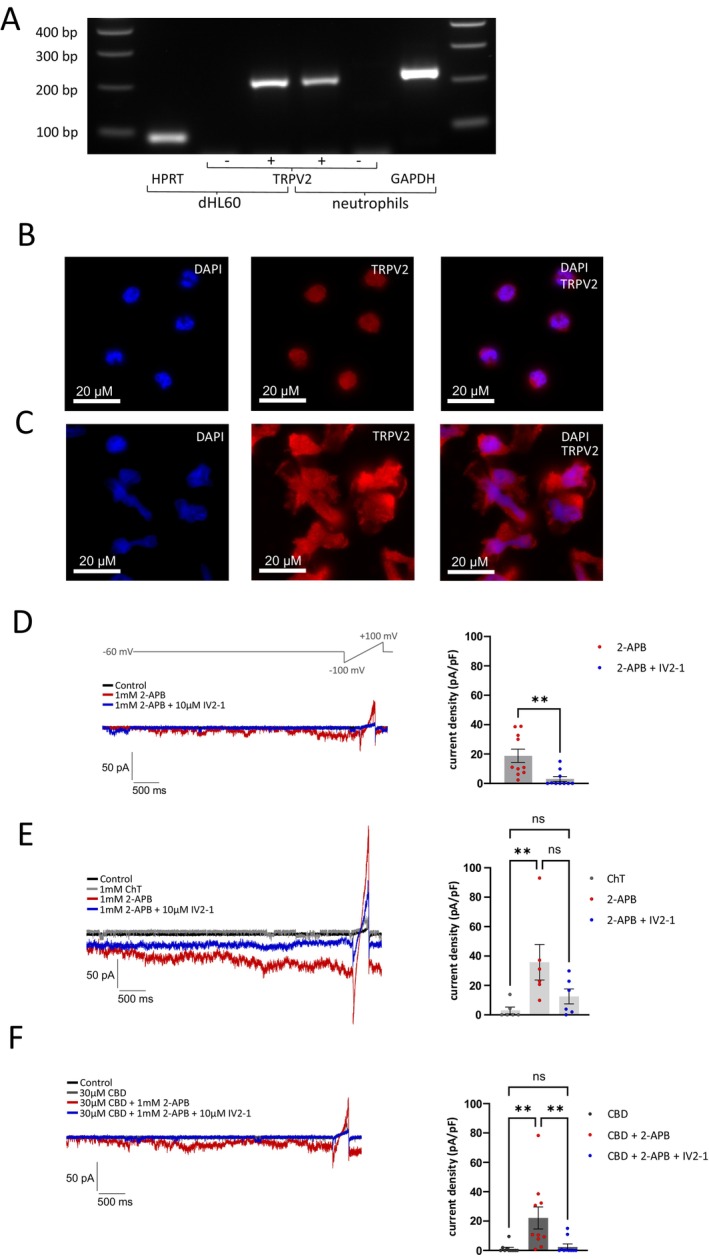
Neutrophils express TRPV2. (A) Visualization of TRPV2‐expression in dHL60 and neutrophils by application of RT‐PCR transcripts specific for TRPV2 (+) on an agarose gel together with RT‐PCR transcripts specific for house‐keeping marker genes for HPRT and GAPDH. Without cDNA synthesis no TRPV2 transcripts were amplified in dHL60 and neutrophil RNA (−). (B, C) Representative immunofluorescence staining of TRPV2 (red) and counterstaining of cell nucleoli with DAPI (blue) in human neutrophils (*n* = 3 human donors, B) and dHL60 (C). (D) Membrane currents with outward currents evoked by voltage ramps in neutrophils evoked by 1 mM 2‐APB. Currents were blocked by 10 μM IV2‐1 (*n* = 10). (Mann–Whitney *U*‐test). (E, F) Membrane currents produced by neutrophils following application of 1 mM ChT (E, *n* = 6) or 30 μM CBD (F, *n* = 10) for 125 s with subsequent application of 1 mM 2‐APB (Kruskal–Wallis test with post hoc Dunn's multiple comparison tests). Experiments for each setting were performed on at least two separate days using neutrophils from at least two individuals. Plotted are means ± SEM, **p < 0.01, ***p < 0.001, ****p < 0.0001. n.s., not significant.

Expression of TRPV2 was detected in human neutrophils from healthy donors as well as in differentiated HL60 cells (dHL60) (Figure 1A). Following differentiation into dHL60, the expression of TRPV2 significantly increased (Figure S1A). Staining confirmed expression of TRPV2 in human neutrophils (Figure 1B) and dHL60 cells (Figure 1C). In order to examine if TRPV2 is functionally expressed in these cells, whole‐cell patch clamp was utilized. Cells were held at −60 mV and 500 ms long voltage‐ramps ranging from −100 to 100 mV were applied to visualize outward rectification of currents (Figure 1D). In agreement with the notion that human TRPV2 is only weakly sensitive to the agonist 2‐APB [6], 1 mM 2‐APB evoked small inward currents at −60 mV that exhibited an outward rectification (Figure 1D). 2‐APB‐induced currents were inhibited by 10 μM of the selective TRPV2‐antagonist 5‐(1,3‐dithiolan‐2‐ylidene)‐4‐methyl‐5‐phenylpentan‐2‐one (IV2‐1). We previously demonstrated that oxidation can sensitize TRPV2 [7]. Indeed, neutrophils treated with 1 mM of the oxidant chloramine T (ChT) for 125 s produced large outwardly rectifying 2‐APB‐induced currents that were partly inhibited by IV2‐1 (Figure 1E). It is possible that the residual current that was not inhibited by IV2‐1 was generated by TRPM2 known to be sensitive to ChT [8]. Cannabidiol (CBD) also strongly sensitizes TRPV2 to 2‐APB [9]. While 30 μM CBD alone did not induce profound currents in neutrophils, the subsequent 2‐APB application evoked robust membrane currents that were blocked by IV2‐1 (Figure 1F). Almost identical effects were observed in experiments on dHL‐60 cells (Figure S1B,C).

**FIGURE 2:**
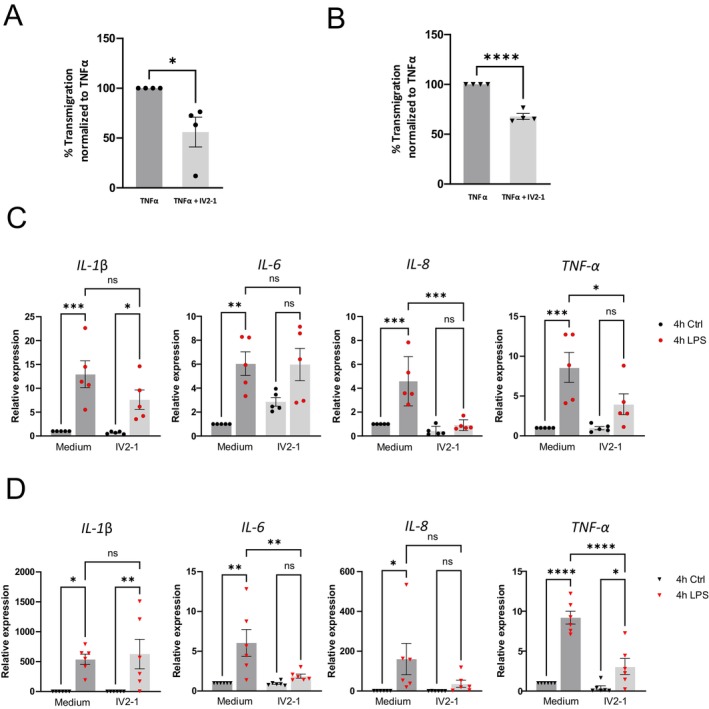
TRPV2 is important for neutrophil function (A, B) Transmigration of neutrophils (A, *n* = 4) and dHL60 (B, *n* = 4) after activation with TNF‐α without (Medium) and with 10 μM IV2‐1. Results are normalized to TNF‐α as 100% (unpaired two‐sided *t*‐tests). (C, D) Relative expression of IL‐1β, IL‐6, IL‐8, and TNF‐α of untreated (Ctrl) and 4 h LPS‐treated (100 ng/mL) neutrophils (C, *n* = 5) and dHL60 (D, *n* = 6) with and without IV2‐1 (one‐way ANOVA with *post hoc* Šidáks multiple comparison tests). Data are given as mean ± SEM, *p < 0.05, **p < 0.01, ***p < 0.001, ****p < 0.0001, n.s., not significant.

TRPV2 was previously found to be important for the migration of macrophages [2]. Accordingly, IV2‐1 significantly diminished the migration rate of tumor necrosis factor‐α (TNF‐α)‐activated neutrophils (Figure 2A) and dHL60 cells (Figure 2B). We also explored the effect of TRPV2 on LPS‐induced cytokine response. Stimulation with LPS increased the expression of interleukin (IL)‐1β, IL‐6, IL‐8, and TNF‐α in both neutrophils (Figure 2C) and dHL60 cells (Figure 2D). While inhibition of TRPV2 significantly reduced the upregulation of IL‐8 and TNF‐α in neutrophils, the expression of IL‐1β or IL‐6 was reduced when TRPV2 was partially blocked by IV2‐1 (Figure 2C). In dHL‐60 cells, inhibition of TRPV2 significantly reduced the upregulation of both IL‐6 and TNF‐α.

The capsaicin‐receptor TRPV1 can also respond to 2‐APB, ChT, and CBD, and it was reported to be expressed in neutrophils [10]. In patch clamp experiments on dHL60 cells, we did not observe any effects at the saturating concentration of 10 μM capsaicin (Figure S1A). Therefore, it seems unlikely that TRPV1 is functionally expressed in these cells.

Taken together, our data show that TRPV2 is functionally expressed in neutrophils and that it plays a relevant role for both migration and cytokine expression. While several TRP channels appear to be relevant for the proper function of neutrophils, TRPM2 may be the best studied [1]. Both TRPM2‐ and TRPV2‐knockout mice display an increased susceptibility to sepsis, and initial reports ascribed these phenotypes as a consequence of a reduced function of macrophages [2, 11]. However, neutrophils lacking TRPM2 also show a marked phenotype following systemic inflammation with increased cytokine levels, a dysregulation of intracellular Ca^2+^ and an impaired formation of neutrophil extracellular traps [12, 13]. Considering that both TRPM2 and TRPV2 are gated by heat and oxidative stress, it seems possible that they fulfill similar or even redundant cellular functions. TRPV2 might influence neutrophil function similar to its involvement in macrophages, where Ca^2+^ influx induces changes in actin polymerization and therefore promotes migration and secretion of signaling molecules [14]. Further studies into the exact involvement of TRPV2 for neutrophil function are warranted, and neutrophils should be taken into account when TRPV2 is discussed as a target for novel anti‐inflammatory drugs.

## 3 Material and Methods

### 3.1 Isolation of Human Primary Neutrophils

Neutrophils were isolated from healthy donors as described previously [15]. Written informed consent was obtained, and collection was approved by the Hannover Medical School Ethics Committee (22614_BO_K_2024).

### 3.2 Cell Culture

Promyelocytic leukemia cell line HL‐60 (ATCC) was cultured in RPMI 1640 (Gibco, Thermo Fisher) supplemented with 15% nonheat inactivated fetal bovine serum (FBS; Biochrom), L‐glutamine, and 1% penicillin and streptomycin (both Bio&Sell) at 37°C and 5% CO_2_. Differentiation into neutrophile‐like cells (dHL60) was achieved by the addition of 1.25% dimethyl sulfoxide (Carl Roth) for 4 to 5 days. The human endothelial Eahy926 cell line (ATCC) was cultured in Dulbecco's Modified Eagle's Medium (DMEM; Bio&Sell) + 10% FBS, L‐glutamine, and 1% penicillin and streptomycin at 37°C and 5% CO_2_.

### 3.3 Immunofluorescence

Cells seeded on coverslips were fixed with 4% paraformaldehyde and permeabilized with 0.1% TritonX100 (Carl Roth). After blocking of unspecific staining with 5% BSA and 5% goat serum for 30 min, primary antibody against TRPV2 (rabbit anti‐human; Thermo Fisher) was diluted 1:100 in PBS supplemented with 1% BSA, and cells were stained at 4°C overnight. Secondary antibody coupled to Cy3 (donkey anti‐rabbit; Jackson ImmunoResearch) was diluted 1:100 in PBS and incubated for 1 h at room temperature. Nuclei were counterstained with 1 μg/mL 4′,6‐Diamidine‐2′‐phenylindole dihydrochloride (DAPI; Applichem) and coverslips were mounted with Fluorescent Mounting Medium (Biozol). Fluorescence was analyzed on an inverted fluorescence microscope (IX‐81, Olympus) with an ORCA‐Flash4.0 camera (Hamamatsu Photonics) using cellSens Dimension 3.2 software (Olympus). Pictures were edited using GIMP (Version GPLv3).

### 3.4 Quantitative Reverse Transcription Polymerase Chain Reaction

Neutrophils and dHL60 cells were stimulated with or without 100 ng/mL LPS (Sigma‐Aldrich) in combination with 10 μM IV2‐1 (MedChemExpress) or blank for 4 h as indicated. RNA was extracted with Tritidy reagent, and 500 ng RNA was used for complementary DNA synthesis (Reverse Transcription Core Kit; Eurogentec). Primers are listed in Table S1. For neutrophils, GAPDH was used as a housekeeper, and for dHL60, HPRT was used. Signals were generated by SYBR green incorporation (SensiFAST SYBR No‐ROX; Meridian Bioscience) into the amplified DNA (40 cycles) on a real‐time polymerase chain reaction cycler (Rotorgene 3000; Corbett Life Science, Qiagen). Data are expressed as 2^−ΔΔCT^. For TRPV2 expression, PCR products were visualized on a 2% agarose gel using ultraviolet light in a Bio‐Vision gel documentation system (Vilber Lourmat).

### 3.5 Patch Clamp

Neutrophils were allowed to adhere on coverslips coated with Poly‐L‐Lysine (Sigma‐Aldrich). dHL60 cells were used after differentiation for 4 to 5 days directly on uncoated coverslips. Whole cell voltage‐clamp was performed using an EPC10 USB amplifier (HEKA). Signals were low passed at 1 kHz and sampled at 2 kHz. Patch pipettes were pulled from borosilicate glass tubes (GB 150 TF‐8P, Science Products) on a Narishige PP‐830 puller to give a resistance of 2 to 5 MΩ. The standard external solution contained 140 mM NaCl, 5 mM KCl, 2 mM MgCl_2_, 5 mM EGTA, 10 mM HEPES, and 10 mM glucose (pH was adjusted with NaOH to 7.4). Pipette solution contained 140 mM KCl, 2 mM MgCl_2_, 5 mM EGTA, and 10 mM HEPES (pH was adjusted with KOH to 7.4). Cells were held at −60 mV. Solutions were applied with a gravity‐driven PTFE/glass multibarrel perfusion system. Combinations of 2‐APB (Tocris Bioscience), IV2‐1, ChT (Carl Roth) and CBD (LGC) or capsaicin (alomone labs) were added to the test solution as indicated. For data acquisition and off‐line analyses, Patchmaster software (HEKA) and Origin 2024b (Origin Lab) were used. Magnitudes of current amplitudes were normalized against the capacitance of each measured cell to give the current density.

### 3.6 Transmigration Assay

Transmigration was performed as described previously [15]. Briefly, Eahy926 cells were cultured on collagen (5 μg/mL rat collagen type I, Corning, Omnilab) coated Transwell filters with 5 μm pores (Costar, Omnilab) for 2 days. On day 3, cells were starved for 6 h in DMEM with 0.5% FBS and stimulated with 10 ng/mL TNF‐α (R&D Systems) for 18 h at 37°C. Control cells remained without stimulation. Cells were diluted to 2.5 × 10^5^ in 100 μL DMEM and stimulated with 10 ng/mL TNF‐α for 30 min with or without 10 μM IV2‐1. Cells were allowed to transmigrate through Eahy926‐covered filters towards 2 mg/mL N‐Formyl‐Met‐Leu‐Phe (Sigma‐Aldrich) for 4 h at 37°C. Migrated cells were visualized by staining nuclei with DAPI and counted using a fluorescence microscope. Whole well surface was counted at 4‐fold magnification and pictures were analyzed using IMAGEJ.

### 3.7 Statistical Analysis

Graphical abstract was created in BioRender. Pantke, S. (2025) https://BioRender.com/1022aih Statistical analyses were performed with GraphPad Prism 10 (GraphPad Software). Values are presented as mean ± SEM, and *p* values < 0.05 were considered significant.

## Author Contributions

Conceptualization: A.S.H., A.L., F.G.E.; Formal Analysis: A.S.H.; Investigation: A.S.H., T.C.F., C.H.; Methodology: A.S.H., C.H., F.G.E.; Supervision: A.L.; Writing: A.S.H., A.L.

## Conflicts of Interest

The authors declare no conflicts of interest.

## Supporting information


**Figure S1:** Expression and function of TRPV2 in dHL60 cells (A) Relative expression of TRPV2 in undifferentiated (*n* = 5) and differentiated (d) HL60 cells (*n* = 11, MWU test). (B) Typical membrane currents with voltage ramps and outward currents at 100 mV in dHL60 cells evoked by 1 mM 2‐APB (*n* = 8, MWU test). (C, D) Illustration of ramps after application of 1 mM ChT (C, *n* = 17) or 30 μM CBD (D, *n* = 20) for 125 s with subsequent application of 1 mM 2‐APB in dHL60 cell with outward currents at 100 mV (Kruskal–Wallis test with post hoc Dunn's multiple comparison tests). Experiments for each setting were performed on at least two separate days. All data are given as mean ± SEM, *p < 0.05, **p < 0.01, ***p < 0.001, ****p < 0.0001.

**Figure S2:** dHL60 cells showed no TRPV1 activation. Measurement of currents by ramps was performed analog to experiments in Figure 2. (A) Representative current trace on dHL60 cells with application of 10 μM Capsaicin (*n* = 7). (B) Current density of outward currents after application of 10 μM Capsaicin (*n* = 7).

**Table S1:** Human primersused for qRT‐PCRs.

## Data Availability

All available data are presented within the manuscript. Original data can be obtained from the corresponding authors upon request.
