# Supplementary material for: TRPV2 Regulates Function of Human Neutrophil Granulocytes
Source: FASEB J. 2025 Sep 15;39(18):e71052. doi: 10.1096/fj.202501585RR (PMC12434796; doi:10.1096/fj.202501585RR)
Supplement: Supplementary file 1 — Figure S1: Expression and function of TRPV2 in dHL60 cells (A) Relative expression of TRPV2 in undifferentiated (n = 5) and differentiated (d) HL60 cells (n = 11, MWU test). (B) Typical membrane currents with voltage ramps and outward currents at 100 mV in dHL60 cells evoked by 1 mM 2‐APB (n = 8, MWU test). (C, D) Illustration of ramps after application of 1 mM ChT (C, n = 17) or 30 μM CBD (D, n = 20) for 125 s with subsequent application of 1 mM 2‐APB in dHL60 cell with outward currents at 100 mV (Kruskal–Wallis test with post hoc Dunn's multiple comparison tests). Experiments for each setting were performed on at least two separate days. All data are given as mean ± SEM, *p < 0.05, **p < 0.01, ***p < 0.001, ****p < 0.0001. [file FSB2-39-e71052-s003.pdf]

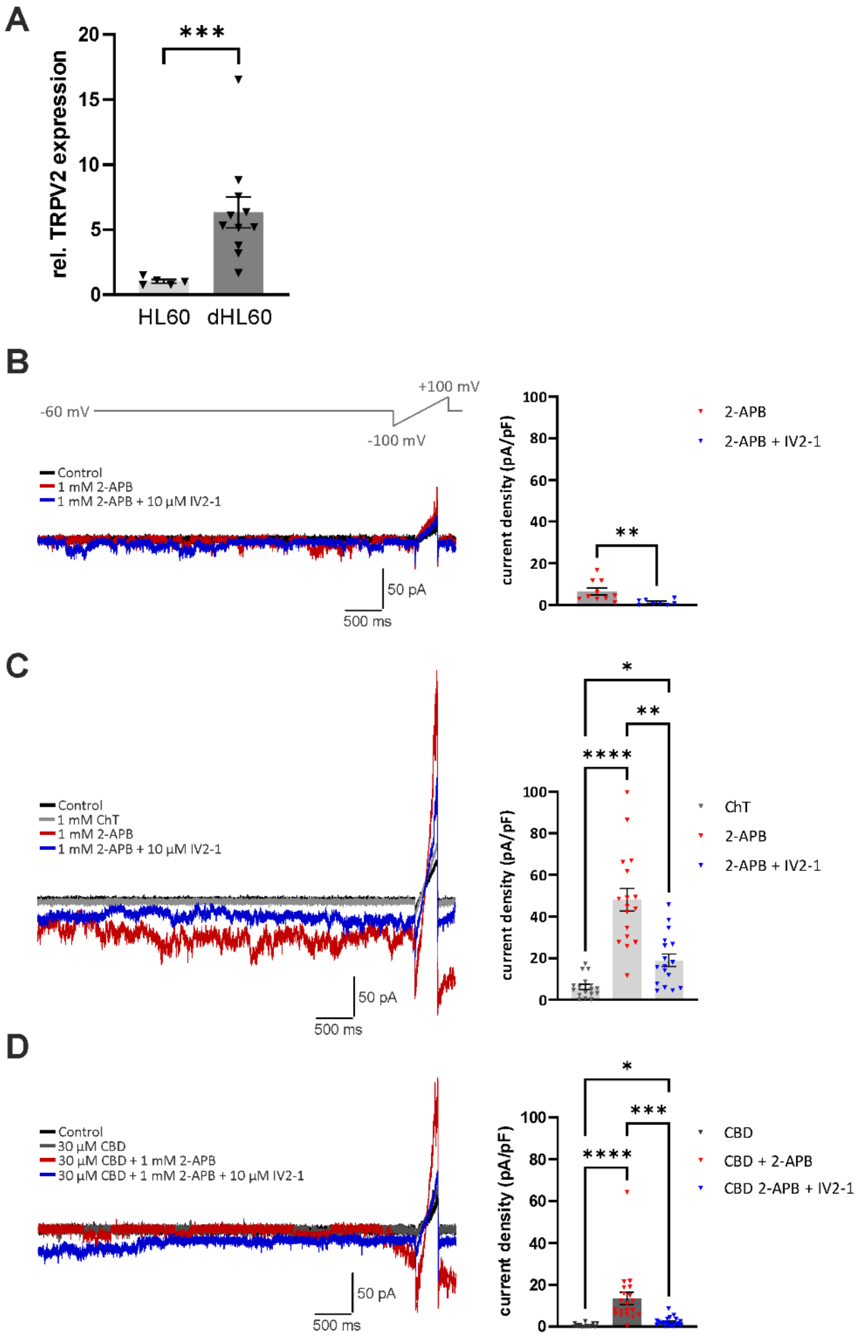

Supplemental Figure S1. Expression and function of TRPV2 in dHL60 cells (A) Relative expression of TRPV2 in undifferentiated (n = 5) and differentiated (d) HL60 cells (n = 11, MWU test). (B) Typical membrane currents with voltage ramps and outward currents at 100 mV in dHL60 cells evoked by 1 mM 2-APB (n = 8, MWU test). (C, D) Illustration of ramps after application of 1 mM ChT (C, *n* = 17) or 30 µM CBD (D, *n* = 20) for 125 seconds with subsequent application of 1 mM 2-APB in dHL60 cell with outward currents at 100 mV (Kruskal-Wallis test with post hoc Dunn's multiple comparison tests). Experiments for each setting were performed on at least two separate days. All data are given as mean ± SEM, *p < 0.05, **p < 0.01, ***p < 0.001, ****p < 0.0001.
