# Supplementary material for: TRPV2 Regulates Function of Human Neutrophil Granulocytes
Source: FASEB J. 2025 Sep 15;39(18):e71052. doi: 10.1096/fj.202501585RR (PMC12434796; doi:10.1096/fj.202501585RR)
Supplement: Supplementary file 2 — Figure S2: dHL60 cells showed no TRPV1 activation. Measurement of currents by ramps was performed analog to experiments in Figure 2. (A) Representative current trace on dHL60 cells with application of 10 μM Capsaicin (n = 7). (B) Current density of outward currents after application of 10 μM Capsaicin (n = 7). [file FSB2-39-e71052-s002.pdf]

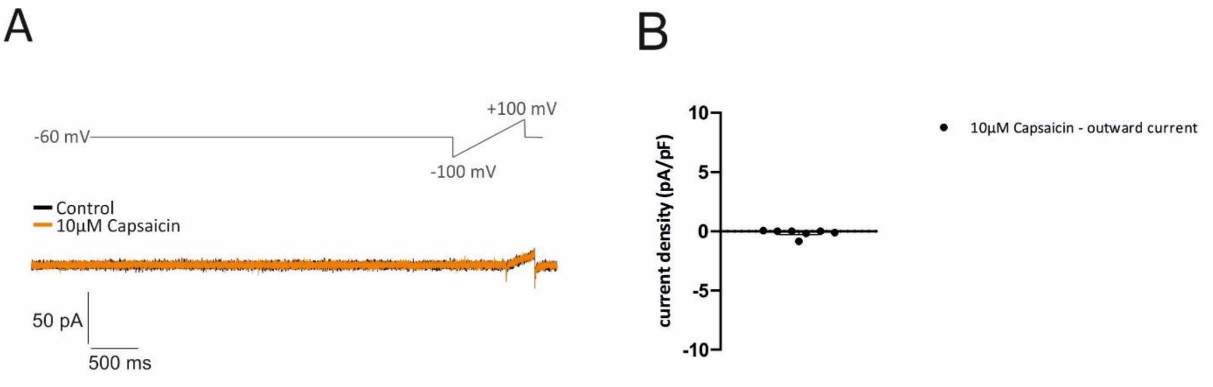

Supplemental Figure 2. dHL60 cells showed no TRPV1 activation. Measurement of currents by ramps was performed analogue to experiments in Fig. 2. (A) Representative current trace on dHL60 cells with application of 10µM Capsaicin (*n* = 7). (B) Current density of outward currents after application of 10µM Capsaicin (*n* = 7)
