# Supplementary material for: TRPV2 Regulates Function of Human Neutrophil Granulocytes
Source: FASEB J. 2025 Sep 15;39(18):e71052. doi: 10.1096/fj.202501585RR (PMC12434796; doi:10.1096/fj.202501585RR)
Supplement: Supplementary file 3 — Table S1: Human primersused for qRT‐PCRs. [file FSB2-39-e71052-s001.pdf]

Supplemental Table 1. Human primers used for qRT-PCRs

Gene	Forward primer (5'-3')	Reverse primer (3'-5')
HPRT	TCAGGCAGTATAATCCAAAGATGG	AGTCTGGCTTATATCCAACACTTCG
GAPDH	TCTCCTCTGACTTCAACAGCGACA	TGAGGGTCTCTCTCTTCCTCTTGT
TRPV2	CCTACGCTGTGTGAGGACC	CCAGCAGATGTGGTTGGAAAG
TNF-α	CTGGGCAGGTCTACTTTGGG	CTGGAGGCCCCAGTTTGAAT
IL1-β	AGCAGAAAACATGCCCGTCT	GACTGTCCTGGCTGATGGAC
IL-6	CCTGACCCAACCACAAATGC	ATCTGAGGTGCCCATGCTAC
IL-8	GGCAGCCTTCCTGATTTCT	CCTACAACAGACCCACACAATA
